# A Pilot Study of Autonomic Function Screening Tests for Differentiating Complex Regional Pain Syndrome Type II and Traumatic Neuropathic Pain

**DOI:** 10.3390/medicina59040646

**Published:** 2023-03-24

**Authors:** Dayoung Kim, Min Jung Kim, Jae Hun Kim, Jeeyoung Oh, Kyomin Choi

**Affiliations:** 1 Department of Neurology, Konkuk University College of Medicine, Konkuk University Medical Center, Seoul 05030, Republic of Korea; 2 Department of Anesthesiology and Pain Medicine, Konkuk University College of Medicine, Konkuk University Medical Center, Seoul 05030, Republic of Korea

**Keywords:** complex regional pain syndrome, neuropathic pain, autonomic function test, QSART, dysautonomia

## Abstract

*Background and Objectives:* One of the most challenging tasks in a clinical setting is to differentiate between complex regional pain syndrome (CRPS) type II and traumatic neuropathic pain (NeP). CRPS is characterized by several dysautonomic manifestations, such as edema, hyper/hypohidrosis, skin color change, and tachycardia. This study compared the outcomes of autonomic function screening tests in patients with CRPS type II and traumatic NeP for diagnostic differentiation. *Materials and Methods:* CRPS type II was diagnosed according to the Budapest research criteria, while NeP was diagnosed according to the updated grading system suggested by the International Association for the Study of Pain Special Interest Group on Neuropathic Pain in 2016. Twenty patients with CRPS type II and twenty-five with traumatic NeP were investigated. *Results:* Twelve patients with CRPS type II presented abnormal results for the quantitative sudomotor axon reflex test (QSART). Abnormal QSART results were more common in the CRPS type II group. *Conclusions*: Analysis of QSART combined with other ancillary tests can help in the differential diagnosis of CRPS type II and traumatic NeP if factors influencing abnormal QSART are sufficiently controlled.

## 1. Introduction

Complex regional pain syndrome (CRPS) is a chronic pain disorder characterized by hyperalgesia and allodynia at the affected site. CRPS can be categorized into CRPS types I and II. CRPS Type II has a history of associated peripheral nerve injury, whereas CRPS type I occurs when there is no confirmed nerve injury [1]. In more advanced diagnostic criteria, a third type known as CRPS-NOS (not otherwise specified) has been proposed, which refers to a clinical presentation that is consistent with CRPS but does not fully meet the diagnostic criteria, and when no other appropriate diagnosis can be made [2,3,4]. The exact pathogenesis of CRPS remains unknown; however, recent studies have suggested that exaggerated inflammation after tissue injury, autoimmunity, and central reorganization in neuronal plasticity are related to the development of this chronic pain disorder [5]. Autonomic imbalance is a commonly accompanying manifestation, and characteristic features include edema, vasodilation, hyper/hypohidrosis, skin color change, tachycardia, and decreased heart rate variability [6].

In South Korea, CRPS patients are registered and supported in the Rare Intractable Disease (RID) program run by the government. Although the diagnosis of CRPS depends on clinical symptoms and signs, a detailed diagnostic evaluation by the Budapest re-search criteria [7,8] and several additional tests have been performed to assess the CRPS status due to registration in the RID program. One of these additional tests is the autonomic function screening test, which consists of tests that evaluate the sudomotor, cardiovascular adrenergic, and parasympathetic functions. The first sudomotor function was evaluated using the quantitative sudomotor axon reflex test (QSART), while cardiovascular adrenergic function is typically measured by recording the changes in blood pressure during the Valsalva maneuver and hemodynamic changes in the head-up tilt (HUT) test. The heart rate response to deep breathing (HRDB) and Valsalva maneuver represent the parasympathetic functions [9]. Abnormalities in the aforementioned tests are utilized in the RID program registration of the CPRS through the detection of the autonomic imbalance. Among them, QSART has been considered the most supportive test for the diagnosis of CRPS; however, a recent study showed a low diagnostic value of QSART [10].

A previous study investigated a large number of patients with chronic pain suspect-ed of CRPS; however, the specificity of the disease was quite heterogeneous [10]. One of the most challenging aspects of diagnosis in a clinical setting is to differentiate between CRPS type II and traumatic neuropathic pain (NeP), as both of these disorders can be diagnosed clinically, and it is difficult to objectively confirm the combined abnormal signs observed in CRPS patients.

Therefore, this study aimed to compare the outcomes of autonomic function screening tests in patients with CRPS type II and traumatic NeP and examine their usefulness for diagnostic differentiation.

## 2. Materials and Methods

### 2.1. Study Design and Population

This retrospective cross-sectional study reviewed the medical records of adults who visited the pain or neurologic clinic for registering in the RID program at a single tertiary center between January 2015 and December 2021. Patients with a history of chronic pain (more than 2 months of symptom period) of the limbs and previous traumatic nerve injury at the affected site were included and classified into CRPS type II or traumatic NeP groups. Participants who had other causes of NeP (e.g., diabetes, Lyme disease, human immunodeficiency virus/acquired immune deficiency syndrome, amyloidosis, vitamin B deficiency, Sjögren syndrome, porphyria, history of alcohol abuse, uremia, monoclonal gammopathy, etc.) and those who underwent insufficient examination were excluded. The enrolled participants were reviewed for clinical symptoms, signs, age, sex, past medical history, adjusted pain medication, affected site, and severity of pain using an 11-point numerical rating scale (NRS).

### 2.2. Diagnosis of CRPS Type II and Traumatic Neuropathic Pain

CRPS type II was diagnosed according to the Budapest research criteria [7] and history of peripheral nerve injury. Clinical signs and symptoms were evaluated based on four categories: positive sensory, vascular, edema, sweating abnormalities, and motor or trophic changes. All enrolled patients with CRPS had to present with ≥1 symptom belonging to each of the four categories and ≥1 sign in each of two or more categories. The auxiliary lab tests performed included a simple X-ray, three-phase bone scan, digital infrared thermal imaging (DITI), quantitative sensory tests, and nerve conduction studies [2].

Patients not diagnosed with CRPS were re-evaluated for NeP, and the diagnoses were based on the updated grading system for NeP, suggested by the International Association for the Study of Pain (IASP) Special Interest Group on Neuropathic Pain (NeuPSIG) in 2016 [11]. All enrolled participants in the traumatic NeP group were diagnosed with “definite” NeP with related trauma history.

The participants’ history, comorbidities, neuroanatomically relevant neurological lesions, clinical symptoms, signs, and outcomes of auxiliary tests were reviewed and determined by pain and neuromuscular specialists.

### 2.3. Autonomic Function Screening Test

All participants underwent an autonomic function screening test as an auxiliary examination for the diagnosis of CRPS. The test consists of the HRDB, Valsalva maneuver, HUT test, and QSART, and was performed according to the Korean guidelines for autonomic function tests [12]. The protocols and instruments used are listed in Appendix A (Table A1). The heart rate variability during deep breathing and the Valsalva maneuver, blood pressure changes on orthostasis, and outcomes of QSART have quantitative outcomes and were evaluated according to the normal reference range in healthy Korean adults and subsequently determined to be normal or abnormal [13]. Interpretations of the sympathetic cardioadrenergic function on the Valsalva maneuver and HUT test also had dichotomous results. The loss of phase produced by the blood pressure change was considered abnormal in the Valsalva maneuver. Orthostatic hypotension and postural tachycardia syndrome were considered abnormal on the HUT test. All reviews and interpretations of autonomic function screening tests were performed by a neuromuscular specialist.

### 2.4. Statistical Analysis

Among the CRPS type II and traumatic NeP group results, the dichotomous results were analyzed using the χ^2^ test or Fisher’s exact test. For the measured values that satisfied the normal distribution through the Kolmogorov–Smirnov test, Student’s t-test was used to analyze the mean value and standard deviation. The group that did not qualify for the normal distribution was analyzed using the Mann–Whitney U test, and the median and quartile values were recorded. We conducted univariable logistic regression analysis to evaluate the impact of each autonomic function screening test item on the traumatic NeP and CRPS type II groups. Subsequently, we performed an additional analysis on the specific autonomic function screening test item that exhibited a significant difference between the CRPS type II and traumatic NeP groups in the aforementioned analysis. A model was created using multivariable logistic regression, incorporating diagnostically and clinically important variables to identify factors that differentiate between groups with and without abnormalities in the selected item. All statistical analyses were performed using SPSS statistical software (version 25.0, IBM, Armonk, NY, USA). For this study, *p*-values less than 0.05 were considered statistically significant.

## 3. Results

**Figure 1:**
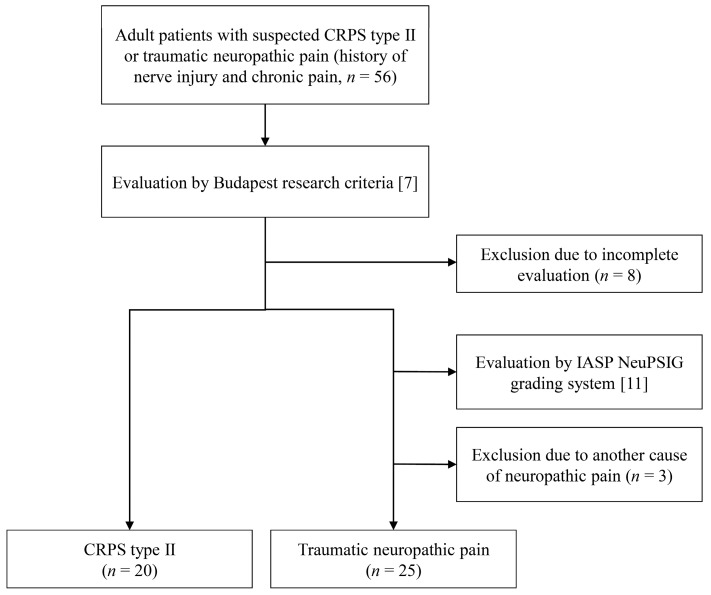
Flowchart of the study population selection. CRPS: complex regional pain syndrome; IASP: International Association for the Study of Pain; NeuPSIG: Special Interest Group on Neuropathic Pain.

Among the 56 patients with chronic pain (more than 2 months) with a history of nerve injury history, 8 were excluded due to deviation from the evaluation process. Twenty patients were diagnosed with CRPS type II, and the remaining twenty-eight patients were re-evaluated using the IASP NeuPSIG grading system [7,11]. All 28 patients were considered as having “definite” NeP; 3 of them were excluded because the disease etiology was metabolic disease, including diabetes mellitus and alcohol abuse. Therefore, 20 patients with CRPS type II and 25 patients with traumatic NeP were finally investigated (Figure 1). The median age of both groups was 40 years (±16.1 in the CRPS type II group and ±14.0 in the traumatic NeP group) (Table 1). There were no significant differences in the body mass index, disease duration, affected site, or etiologies of both the groups. The severity of pain measured by NRS was significantly more severe in the CRPS type II group than in the traumatic NeP group (7.5 ± 1.54, 5.8 ± 1.85, respectively, *p* = 0.001). Bilateral symptoms were more common in the traumatic NeP group than in the CRPS type II group (ten patients, one patient, respectively; *p* = 0.012). Patients with psychiatric disorders were more common in the CRPS type II group than in the traumatic NeP group (ten patients, four patients, respectively, *p* = 0.014). There were no differences in the adjustment of opioids and anticonvulsants, whereas antidepressants were more commonly used in the CRPS type II group than in the traumatic NeP group (16 and 12 patients, respectively, *p* = 0.035).

In the autonomic function screening test, there were no significant differences in abnormal outcomes between the HRDB and Valsalva maneuver tests in the CRPS type II and traumatic NeP groups (Table 2). Twelve of the twenty CRPS type II patients revealed abnormal results in QSART; abnormal QSART outcomes were more common in the CRPS type II group than the traumatic NeP group (six of twenty-five patients, OR = 4.750, 95% CI 1.318 to 17.113, *p =* 0.017). Although not statistically significant, abnormal HUT test outcomes were more frequently observed in the CRPS type II group compared to the traumatic NeP group (OR = inf, 95% CI 0 to inf, *p* = 0.998).

In the univariable logistic regression analysis, NRS, body mass index (BMI), use of antidepressants, and symptom site did not have a significant effect on QSART results, while the diagnosis of CRPS type II and the presence of a psychiatric disorder did (Table 3). In the multivariable logistic regression analysis using the same parameters, the odds of having abnormal QSART results were significantly higher (OR = 10.920, 95% CI 1.251 to 95.302, *p* = 0.031) in the group diagnosed with CRPS type II compared to the traumatic NeP group (Table 3). A BMI of 1 significantly decreased the odds of abnormal QSART (OR = 0.795, 95% CI 0.638 to 0.992, *p* = 0.042).

Among 20 CRPS type II patients, 13 (65.00%) had an abnormal outcome in the autonomic screening function test (Appendix B, Table A2). Among them, 12 patients simultaneously showed abnormal QSART and HUT test results, and 1 male patient (No. 5) presented with postural tachycardia syndrome on HUT with a normal QSART outcome. Among the 12 patients with abnormal QSART outcomes, 2 patients had excessive sweating and the other 10 patients showed decreased function. The abnormal sweating areas presented by the 12 patients were the same as the pain area, and 3 patients (Nos. 3, 10, and 12) showed abnormalities in sudomotor function in a larger area than the pain site. Among the 13 patients with abnormal autonomic function test results, 12 patients were in a hypothermic state as per the DITI test results, and 8 patients showed an increased uptake in their pain area in the three-phase bone scan test. Three patients (Nos. 7, 10, and 13) showed hypothermia in the non-lesioned limbs on the DITI test.

## 4. Discussion

Autonomic dysfunction in CRPS is thought to be due to abnormal changes in the expression of α-1 adrenergic receptors on keratinocytes and nociceptors [14]. In the acute phase of CRPS, sympathetic nervous system activity decreases, leading to lower circulating levels of norepinephrine, while the peripheral α-1 adrenergic receptors are upregulated and sensitized for compensation [14]. This causes vasodilation and erythema in the CRPS-affected limb [15]. In contrast, during the chronic phase of CRPS, the prolonged release of proinflammatory cytokines results in excessive sympathetic nervous system outflow, leading to increased norepinephrine levels and decreased α-1 adrenergic receptor expression. This leads to vasoconstriction in cold limbs [16]. Higher circulating catecholamines, including norepinephrine and epinephrine, result in an increase in the heart rate and reduced heart rate variability in patients with CRPS compared with that in healthy controls [17,18]. Therefore, these dysautonomic features of CRPS were measured by laboratory examination and were used for the correct and early diagnosis of CRPS.

In this study, the outcomes of HRDB, Valsalva maneuver, HUT test, and QSART were used to evaluate the autonomic dysfunction in patients with type II CRPS and traumatic NeP. Sixty percent (12 patients) of patients with CRPS type II showed abnormalities in the QSART; a significantly higher rate of abnormalities was observed in the CRPS group than the traumatic NeP group. Although abnormal results in the HUT test were also higher in CRPS type II, only four abnormal HUT test results were observed in twenty patients with CRPS type II. These abnormal results were infrequent and not significant in the CRPS group, indicating that the diagnostic performance of these autonomic function screening test items would be insufficient in a clinical setting. However, QSART showed a relatively higher rate of abnormalities in CRPS type II patients and an association with affected pain sites. These results are similar to those of previous studies, suggesting that the full autonomic function screening test may be difficult to use in the early diagnosis of CRPS, and that only the QSART is most likely to be used in differential diagnosis [10,19]. Considering the results of this study, the QSART and confirmation of bilateral lesions may contribute to the differentiation between CRPS type II and traumatic NeP. Psychiatric disorders also have been found to be significantly common in CRPS. This should be taken into consideration when diagnosing CRPS in a clinical setting.

As abnormalities in QSART may have clinical significance in the diagnosis of CRPS type II, we aimed to identify factors that could potentially affect the QSART results. Previous studies have reported an association between decreased cardiac vagal tone during autonomic function tests and overweight or obesity [20]. Additionally, there have been reports of a correlation between QSART values and BMI in adults [13]. In the present study, a multiple regression analysis model revealed that a decrease in BMI had a significant effect on QSART results. However, given the limited sample size and the fact that not all relevant variables were included in the analysis, the interpretation of the findings requires caution. The results of this study, along with previous research, indicate the necessity for follow-up studies to investigate the factors that influence autonomic function test results.

This study had several limitations. First, this retrospective cross-sectional study may not sufficiently reflect the natural course of CRPS type II and traumatic NeP. As both diseases have a dynamic course, a cross-sectional study might be inappropriate for evaluating the characteristics of the disease. In particular, patients with traumatic NeP may develop CRPS over time. Second, this study had a small number of participants, and it is difficult to generalize these findings due to the single-center study design with strict enrollment criteria for CRPS and NeP. As mentioned earlier, to register in a government program, not only clinical standards but also more stringent standards may be required. This clinical environment may have influenced the review of medical records. Nevertheless, this may also suggest that the classification of the participant groups was properly determined. Third, although there are several diagnostic criteria for CRPS, only the Budapest research criteria were used in this study. This result reflects the current situation in which the criteria are being used for registration in South Korea’s RID program. These limitations suggest that further studies are needed, such as repeated autonomic function screening tests in patients with nerve injury that can develop into CRPS.

This study was conducted in more specific patients, such as those with CRPS type II and traumatic NeP, compared to previous studies The analysis of the QSART combined with auxiliary tests can be helpful in the differential diagnosis of CRPS type II with traumatic NeP.

## 5. Conclusions

Abnormal QSART test results were significantly more common in the CRPS type II group than in the traumatic NeP group. Analysis of QSART and other ancillary tests can help in the differential diagnosis of CRPS type II and traumatic NeP if factors influencing abnormal QSART are sufficiently controlled.

## Figures and Tables

**Table 1:** Clinical Characteristics of participants with CRPS type II and traumatic neuropathic pain.

	CRPS Type II (*n* = 20)	Traumatic Neuropathic Pain (*n* = 25)	*p*-Value
Age * (years)	40 ± 16.1	40 ± 14.0	0.87
Sex ^†^			0.55
Male	13 (65.00)	13 (52.00)	
Female	7 (35.00)	12 (48.00)	
BMI * (kg/m^2^)	25.1 ± 3.00	24.0 ± 4.70	0.31
Duration ^‡^ (years)	3.0 (1.00–6.25)	4.0 (3.00–6.50)	0.27
NRS * (0–10)	7.5 ± 1.54	5.8 ± 1.85	0.001 ^§^
Affected site ^†^			
Upper extremity	8 (40.00)	6 (24.00)	0.34
Lower extremity	12 (60.00)	15 (60.00)	1.00
Upper or lower	0 (0.00)	4 (16.00)	0.12
Bilateral symptom	1	10	0.012 ^§^
Psychiatric disorders ^†^	10 (50.00)	4 (16.00)	0.014 ^§^
Etiology ^†^			
Contusion/laceration	9	11	0.95
Traffic accident	2	7	0.26
Surgery	2	2	1.00
Fracture	2	1	0.58
Combined ^‖^	5	4	0.48
Medication ^†^			
Opioids	19 (95.00)	20 (80.00)	0.21
Anticonvulsants	19 (95.00)	22 (88.00)	0.62
Antidepressants	16 (80.00)	12 (48.00)	0.035 ^§^

CRPS: complex regional pain syndrome; BMI: body mass index; NRS: numerical rating scale * The data are presented as the mean ± standard deviation. ^†^ Data are presented as the number (%). ^‡^ Data are presented as the median (Q1–Q3). ^§^ *p* < 0.05. ^‖^ Categorized as “combined” if there is more than one of the above causes.

**Table 2:** Comparison of autonomic function screening test between the CRPS type II group and the traumatic neuropathic pain group.

	CRPS Type II * (*n* = 20)	Traumatic Neuropathic Pain * (*n* = 25)	OR (95% CI)	*p*-Value
Abnormal QSART	12 (60.00)	6 (24.00)	4.750 (1.318, 17.113)	0.017 ^†^
Abnormal HRDB test	4 (20.00)	3 (12.00)	1.833 (0.359, 9.353)	0.466
Abnormal Valsalva maneuver test	1 (5.00)	0 (0.00)	Inf (0, Inf)	0.998
Abnormal HUT test	4 (20.00)	0 (0.00)	Inf (0, Inf)	0.998

CRPS: complex regional pain syndrome; QSART: quantitative sudomotor axon reflex test; HRDB: heart rate response to deep breathing; HUT: head-up tilt; OR: odds ratio; CI: confidence interval; Inf: infinity. * Data are presented as number (%). ^†^ *p* < 0.05.

**Table 3:** Result of the univariable and multivariable logistic regression analysis of variable factors for abnormal QSART.

	Univariable Model		Multivariable Model	
	OR (95% CI)	*p*-Value	OR (95% CI)	*p*-Value
CRPS type II	4.750 (1.318, 17.113)	0.017 *	10.920 (1.251, 95.302)	0.031 *
NRS	1.010 (0.737, 1.385)	0.949	0.738 (0.472, 1.1152)	0.181
BMI	0.888 (0.758, 1.040)	0.140	0.795 (0.638, 0.992)	0.042 *
Antidepressants	2.080 (0.578, 7.486)	0.262	0.990 (0.158, 6.195)	0.992
Psychiatric disorders	4.400 (0.059, 0.868)	0.030 *	3.245 (0.550, 19.141)	0.194
Bilateral symptom	0.475 (0.107, 2.107)	0.327	0.822 (0.121, 5.593)	0.841

CRPS: complex regional pain syndrome; NRS: numerical rating scale; BMI: body mass index; OR: odds ratio; CI: confidence interval; Inf: infinity. * *p* < 0.05.

## Data Availability

The data presented in this study are available upon request from the corresponding authors. The data are not publicly available for ethical reasons.

## Appendix A

**Table A1:** Summary of the performed autonomic function screening test [12].

Preparation	▪ Anticholinergic drugs, antihistamines, and cold medications should be discontinued at least 48 h before the test. ▪ Vasoactive drugs with short half-lives (alpha-and beta-agonists and antagonists) should be discontinued before 24 h. ▪ Coffee or cigarettes should be avoided 4 h prior to the test. ▪ Do not eat overnight or at least 4 h prior to the test. ▪ On the day of the examination, do not wear compression garments, including stockings and corsets.
Tests (Perform the following four tests in order)	
QSART	▪ Evaluate the postganglionic sympathetic cholinergic function. ▪ For sweat gland stimulation, iontophoresis using a cholinergic drug, such as acetylcholine, is used. ▪ Basic recording sites: medial forearm, proximal leg, distal leg, and proximal foot (may vary depending on the affected site). ▪ Latency and sweat secretion are obtained at each test site. ▪ Based on the age- and gender-specific normal range of the Korean population, those in the lower 5% or upper 5% were considered as functional abnormalities.
HRDB test	▪ Check for abnormalities in the parasympathetic function to the heart. ▪ Using the dynamic principle (heart rate increase during inhalation and decrease during expiration) caused by breathing. ▪ Take eight regular breaths at a breathing rate of six per minute (five seconds of inhalation and five seconds of exhalation). ▪ Based on the age- and gender-specific normal range of the Korean population, those in the lower 5% were considered as functional abnormalities.
Valsalva maneuver	▪ Cardiovascular adrenergic system (sympathetic nervous system) and cardiovagal nervous system (parasympathetic nervous system) functions can be assessed simultaneously. ▪ Blow the mouthpiece to hold for 15 s at 40 mmHg (at least 30 mmHg but not over 50). ▪ Calculate the ratio of maximum heart rate and minimum heart rate immediately after the Valsalva maneuver. ▪ Check the blood pressure change during the Valsalva maneuver. ▪ Based on the age- and gender-specific normal range of the Korean population, those in the lower 5% in heart rate change were considered as functional abnormalities. ▪ Loss of phases produced by blood pressure was considered as a functional abnormality.
HUT test	▪ Used to evaluate orthostatic intolerance and syncope, such as OH or POTS. HUT is performed at the end of the autonomic nerve test. ▪ Raise the patient with the tilt table to 70° in one smooth continuous motion. ▪ Heart rate and blood pressure were measured at 1, 3, and 5 min. ▪ OH and POTS were considered as functional abnormalities.

QSART: quantitative sudomotor axon reflex test; HRDB: heart rate response to deep breathing; HUT: head-up tilt; OH: orthostatic hypotension; POTS: postural tachycardia syndrome. All tests were performed by FULL AUTONOMIC TESTING LAB, WR Medical Electronics, MN, USA.

## Appendix B

**Table A2:** Comparison of auxiliary tests outcome in CRPS type II patients with abnormal autonomic function screening test.

Patient No.	Onset Age/Sex	Disease Duration (Months)	Affected Site	QSART	HUT Test	HRDB Test	Valsalva Maneuver	DITI	Three-Phase Bone Scan
1	43/M	70	Lower limb, R	Decreased on lower limb, R	N	Normal	N	N	N
2	27/M	91	Upper limb, L	Decreased on upper limb, L	N	Normal	N	Hypothermia on upper limb, L	N
3	24/M	13	Lower limb, R; Sacral area	Increased on whole limbs *	N	Decreased	N	Hypothermia on lower limb, R	Increased uptake on lower limb, R
4	24/M	11	Lower limb, R	Decreased on lower limb, R	OH	Decreased	N	Hypothermia on lower limb, R	Increased uptake on lower limb, R
5	28/M	25	Ankle, R	N	POTS	Normal	N	Hypothermia on ankle, R	ND
6	52/M	38	Foot, L	Decreased on foot, L	N	N	N	Hypothermia on foot, L	Increased uptake on foot, L
7	64/M	15	Upper limb, R	Decreased on upper and lower limb, R	N	N	N	Hypothermia on hand, L ^†^	Increased uptake on upper limb, R
8	26/F	16	Shoulder, L	Decreased on upper limb, L	N	Decreased	Decreased Valsalva ratio	Hypothermia on upper limb and shoulder, L *	N
9	48/F	50	Foot, L	Decreased on foot, L	OH	N	N	Hypothermia on lower limb, L	N
10	44/F	14	Hand, L	Decreased on lower limbs, R, L *	N	Decreased	N	Hypothermia on shoulder, L ^†^	Increased uptake on hand, L
11	58/F	26	Lower limb, R	Decreased on lower limb, R	POTS	N	N	Hypothermia on lower limb, R	Increased uptake on lower limb, R
12	61/F	13	Upper limb, R	Increased on upper limbs, R, L *	N	N	N	Hypothermia on upper limb, R	Increased uptake on upper limb, R
13	43/F	30	Upper limb, R	Decreased on upper limb, R	N	N	N	Hypothermia on upper limb, L ^†^	Increased uptake on upper limb, R

CRPS: complex regional pain syndrome; QSART: quantitative sudomotor axon reflex test; HUT: head-up tilt; HRDB: heart rate response on deep breathing; DITI: digital infrared thermal imaging; R: right; L: left; N: normal; OH: orthostatic hypotension; POTS: postural tachycardia syndrome; ND: not done. * Abnormal outcome was observed in both pain lesion and non-lesioned areas. ^†^ Abnormal outcome was observed in non-lesioned areas.
